# Experimental SARS-CoV-2 Infection of Bank Voles

**DOI:** 10.3201/eid2704.204945

**Published:** 2021-04

**Authors:** Lorenz Ulrich, Anna Michelitsch, Nico Halwe, Kerstin Wernike, Donata Hoffmann, Martin Beer

**Affiliations:** Friedrich-Loeffler-Institut, Greifswald–Insel Riems, Germany

**Keywords:** coronavirus, 2019 novel coronavirus disease, SARS-CoV-2, severe acute respiratory syndrome coronavirus 2, zoonoses, coronavirus disease, COVID-19, viruses, respiratory infections, bank vole, susceptibility, serology, wildlife cycle, sylvatic cycle, rodent, Germany

## Abstract

After experimental inoculation, severe acute respiratory syndrome coronavirus 2 infection was confirmed in bank voles by seroconversion within 8 days and detection of viral RNA in nasal tissue for up to 21 days. However, transmission to contact animals was not detected. Thus, bank voles are unlikely to establish effective transmission cycles in nature.

Severe acute respiratory syndrome coronavirus 2 (SARS-CoV-2) led to a global pandemic in the human population within months after its first reporting (*1*). Potential wildlife reservoirs of SARS-CoV-2 remain unknown; susceptibility of various animal species has been described (*2*,*3*). Among rodent species, the Syrian hamster (*Mesocricetus auratus*) (*4*) and the North American deer mouse (*Peromyscus maniculatus*) (A. Fagre et al., unpub. data, https://doi.org/10.1101/2020.08.07.241810; B.D. Griffin et al., unpub. data, https://doi.org/10.1101/2020.07.25.221291), both *Cricetidae* species, have proved to be highly susceptible. These rodents transmit SARS-CoV-2 to co-housed contact animals and therefore are likely develop effective infection chains in nature, which could result in independent SARS-CoV-2 transmission cycles in nature and sequential reintroduction to the human population (*4*; B.D. Griffin et al., unpub. data, https://doi.org/10.1101/2020.07.25.221291). In Europe, bank voles (*Myodes glareolus*) are a widespread *Cricetidae* species (*5*). We aimed to characterize SARS-CoV-2 infection in bank voles and their ability to maintain sustainable infection chains.

We intranasally inoculated 9 bank voles with SARS-CoV-2 strain Muc-IMB-1 and, 24 hours later, co-housed 1 contact animal with each of 3 groups of 3 inoculated animals (donor–recipient ratio [d:r] 3:1). We took swab samples regularly from all animals (Appendix); we euthanized 1 or 2 animals at predefined times (Appendix). One bank vole did not survive initial anesthesia for inoculation.

Neither inoculated nor contact animals showed clinical signs during the study. We detected seroconversion for all directly inoculated animals euthanized 8, 12, and 21 days postinfection (dpi), whereas the animals euthanized 4 dpi and the contact animals were all clearly seronegative for SARS-CoV-2 antibodies in an already validated indirect multispecies ELISA based on the receptor-binding domain (*6*).

All directly inoculated bank voles tested positive for SARS-CoV-2 by quantitative reverse transcription PCR (qRT-PCR) by oral and rhinarium swab specimens at 2 dpi. At 4 dpi, 5 of these 8 animals were positive by oral swab specimen; 2 were also positive by rhinarium swab specimen. On both those sampling days, rectal swab specimens of 2 animals tested positive for SARS-CoV-2 by qRT-PCR. Groupwise collected fecal samples also tested positive by qRT-PCR at 2 and 4 dpi. All swabs collected 8, 12, and 16 dpi from directly inoculated animals and every swab from the co-housed contact animals tested negative by qRT-PCR (Table; Figure).

**Table:** RT-qPCR results of the swap sampling of all inoculated and contact bank voles*

Box	Status	Swab	−1 dpi	2 dpi	4 dpi	8 dpi	12 dpi	16 dpi
Box 1	Inoculated	Oral	Neg	32.45	Neg	Neg	Neg	Neg
		Nasal	Neg	32.29	Neg	Neg	Neg	Neg
		Rectal	Neg	Neg	Neg	Neg	Neg	Neg
	Inoculated	Oral	Neg	NA	NA	NA	NA	NA
		Nasal	Neg	NA	NA	NA	NA	NA
		Rectal	Neg	NA	NA	NA	NA	NA
	Inoculated	Oral	Neg	32.09	28.16	Neg	Neg	Neg
		Nasal	Neg	31.72	34.03	Neg	Neg	Neg
		Rectal	Neg	36.54	36.39	Neg	Neg	Neg
	Contact	Oral	Neg	Neg	Neg	Neg	Neg	Neg
		Nasal	Neg	Neg	Neg	Neg	Neg	Neg
		Rectal	Neg	Neg	Neg	Neg	Neg	Neg
	Collected feces		Neg	36.58	37.66	Neg	Neg	Neg
Box 2	Inoculated	Oral	Neg	29.40	32.41	NA	NA	NA
		Nasal	Neg	32.68	34.72	NA	NA	NA
		Rectal	Neg	Neg	Neg	NA	NA	NA
	Inoculated	Oral	Neg	30.46	32.54	Neg	NA	NA
		Nasal	Neg	32.30	Neg	Neg	NA	NA
		Rectal	Neg	36.67	Neg	Neg	NA	NA
	Inoculated	Oral	Neg	32.72	37.07	Neg	Neg	Neg
		Nasal	Neg	34.74	Neg	Neg	Neg	Neg
		Rectal	Neg	Neg	Neg	Neg	Neg	Neg
	Contact	Oral	Neg	Neg	Neg	Neg	Neg	Neg
		Nasal	Neg	Neg	Neg	Neg	Neg	Neg
		Rectal	Neg	Neg	Neg	Neg	Neg	Neg
	Collected feces		Neg	36.06	36.65	Neg	Neg	Neg
Box 3	Inoculated	Oral	Neg	30.98	Neg	Neg	NA	NA
		Nasal	Neg	31.63	Neg	Neg	NA	NA
		Rectal	Neg	Neg	Neg	Neg	NA	NA
	Inoculated	Oral	Neg	30.66	34.32	NA	NA	NA
		Nasal	Neg	34.52	Neg	NA	NA	NA
		Rectal	Neg	Neg	34.89	NA	NA	NA
	Inoculated	Oral	Neg	32.64	Neg	Neg	Neg	NA
		Nasal	Neg	35.46	Neg	Neg	Neg	NA
		Rectal	Neg	Neg	Neg	Neg	Neg	NA
	Contact	Oral	Neg	Neg	Neg	Neg	Neg	Neg
		Nasal	Neg	Neg	Neg	Neg	Neg	Neg
		Rectal	Neg	Neg	Neg	Neg	Neg	Neg
	Collected feces		Neg	36.62	37.02	Neg	Neg	Neg

*Results are given in quantification cycle values. dpi, days postinoculation; NA, not applicable; Neg, negative.

**Figure:**
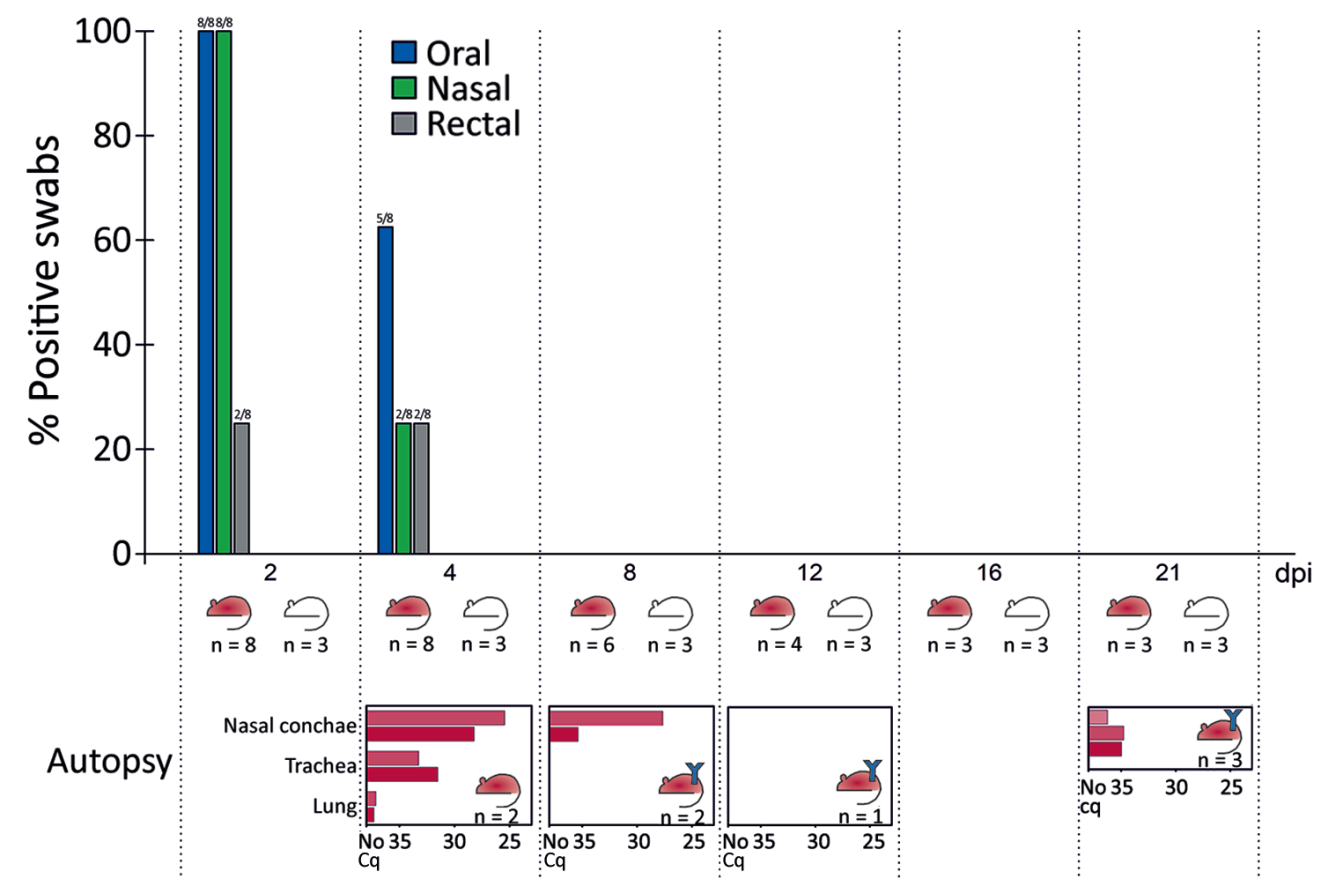
Percentage of SARS-CoV-2–positive swab specimens on all sampling time points in experimental SARS-CoV-2 infection of bank voles. The red mouse symbols symbolize inoculated bank voles; the white mouse symbols represent co-housed contact bank voles. Blue Y symbols stand for detected antibodies against SARS-CoV-2 in the respective bank vole group. Quantitative reverse transcription PCR results for the sampled organs of the euthanized, inoculated bank voles are given below the main chart for each time point. Cq, quantification cycle; dpi, days postinoculation; n, number of bank voles; SARS-CoV-2, severe acute respiratory syndrome coronavirus 2.

Two animals were euthanized at 4 dpi; nasal conchae, trachea, lung, and olfactory bulb samples tested positive for SARS-CoV-2 RNA by qRT-PCR (quantification cycle [Cq] 25.45–37.15). One animal showed viral genome in cerebrum and cerebellum samples, whereas the spleen sample from the other animal was positive for the viral genome. At 8 dpi another 2 animals were euthanized; both exhibited viral RNA only within the nasal conchae. The animal euthanized at 12 dpi was negative in all collected tissue samples. Nasal conchae of 3 inoculated animals euthanized at 21 dpi tested positive by qRT-PCR in the (Cq values 34.78, 34.97, 36.25), whereas all 3 contact animals euthanized at the same time tested negative in the nasal conchae.

Reisolation of viable virus from tissue materials in cell culture (Vero E6) was successful for 1 nasal conchae sample taken at 4 dpi. However, isolation from samples with Cq >28 failed, in line with findings of other groups (*3*,*7*).

Overall, bank voles proved to be susceptible to infection with SARS-CoV-2 but did not transmit the virus to co-housed direct contact animals (initial d:r 3:1), in contrast to highly susceptible hamsters or deer mice, which transmit SARS-CoV-2 to each contact animal (d:r 1:1) within 5 days (*4*; B.D. Griffin et al., unpub. data, https://doi.org/10.1101/2020.07.25.221291). Our results suggest a tissue tropism for SARS-CoV-2 replication in bank voles to the upper respiratory tract, as seen for other species, such as ferrets, fruit bats, and raccoon dogs (*3*,*7*). The persistence of viral genome for at least 3 weeks in nasal tissue of directly inoculated animals was unexpected, especially because the last positive sample was retrieved 4 dpi from the respective bank voles (Table). This finding is most likely the result of the suspected clustering of SARS-CoV-2 infection foci in narrow areas of the upper respiratory tract (L.M. Zaeck et al., unpub. data, https://doi.org/10.1101/2020.10.17.339051). Considering that virus isolation from these 21 dpi samples was not successful, the persistence of SARS-CoV-2 is unlikely to lead to the same shedding of infectious virus as it was shown previously for deer mice (A. Fagre et al., unpub. data, https://doi.org/10.1101/2020.08.07.241810; B.D. Griffin et al., unpub. data, https://doi.org/10.1101/2020.07.25.221291). Deer mice also seem to shed virus through the rectum. However, in bank voles, the SARS-CoV-2 genome could not be detected in the intestines. Although rectal swabs and fecal samples were RT-qPCR positive, the detected Cq values were high, indicating low viral RNA levels. Therefore, the detected viral RNA likely represents residues, which might have resulted from extensive grooming behavior and therefore do not correspond with actual virus shedding from the rectum or feces.

This study proves a general susceptibility of bank voles toward SARS-CoV-2 infection. However, bank voles did not transmit SARS-CoV-2 to contact animals, making them unlikely to maintain sustainable infection chains in nature. Therefore, the risk of bank voles becoming a reservoir for SARS-CoV-2 in nature (for example, after contact with infected cats) is low.

Appendix Additional information on the experimental SARS-CoV-2 infection of bank voles.
