# Supplementary material for: Experimental SARS-CoV-2 Infection of Bank Voles
Source: Emerg Infect Dis. 2021 Apr;27(4):1193–5. doi: 10.3201/eid2704.204945 (PMC8007283; doi:10.3201/eid2704.204945)
Supplement: Appendix — Additional information on the experimental SARS-CoV-2 infection of bank voles. [file 20-4945-Techapp-s1.pdf]

Article DOI: https://doi.org/10.3201/eid2704.204945

Experimental SARS-CoV-2 Infection of Bank Voles

Appendix

Animals and Housing Conditions

We obtained 8 female and 4 male bank voles, 7–9 weeks of age, from an in-house breeding colony at the Friedrich-Loeffler-Institut, Insel Riems, Germany. Prior to infection, we determined negative serologic status toward SARS-CoV-2 of the breeding colony by an indirect receptor-binding domain (RBD)-ELISA (*1*). All animals used in the trial tested RT-qPCR negative for SARS-CoV-2 on the day before infection by rhinarium, oral, and rectal swabs. For the duration of the study the animals were kept in individually ventilated cages (IVCs) with a light regime of 12 hours illumination and 12 hours darkness. Drinking water and a rodent diet were provided ad libitum. All handling procedures were performed under biosafety level 3 (BSL-3) conditions.

Study Design

We inoculated 9 bank voles with $1{\times}10^5$ tissue culture infection dose 50 ($TCID_{50}$) of the SARS-CoV-2 strain 2019_nCoV Muc-IMB-1 (GISAID ID_EPI_ISL_406862, designation hCoV-19/Germany/BavPat1/2020) by administering 70 μL virus suspension to the nostrils and rhinarium. Inoculation took place under a short-term isoflurane-based inhalation anesthesia. Three inoculated bank voles were housed together in 1 IVC. Twenty-four hours after inoculation another 3 naïve in-contact bank voles, 1 per IVC, were co-housed with the directly inoculated animals. Physical examinations following a defined clinical score regarding general behavior, respiration, eyes, and neurologic symptoms were performed daily and bodyweight changes were monitored regularly (0, 2, 3, 4, 6, 7, 8, 9, 10, 12, 14, 16, and 21 days postinfection [dpi]). Oral, rhinarium, and cloacal swabs were taken from each animal at 2, 4, 8, 12, and 16 dpi. A fecal sample was taken from each IVC at these sampling points.

Two bank voles each were euthanized at 4 and 8 dpi and another at 12 dpi. At autopsy, a serum sample was collected and the nasal conchae, trachea, lung, heart, olfactory bulb, forebrain, cerebellum, liver, spleen, kidney, and small and large intestines were sampled. The remaining animals were euthanized at 21 dpi and serum samples were collected, as well as a sample of the nasal conchae.

Antibody Detection

Serum samples taken during euthanasia were tested by RBD-ELISA (*1*). Absorbance values >0.3 were considered antibody positive, those <0.2 antibody negative, and those in between as questionable (Appendix Table).

RNA Extraction and RT-qPCR

Before sampling, swabs (nerbe plus GmbH, https://www.nerbe-plus.de; Copan Italia S.p.A., https://www.copangroup.com) were dampened with Hank's 692 balanced salts (HBS) and Earle's balanced salts (EBS) in minimum essential medium (MEM). After sampling, the swabs were resuspended in 1 mL HBS and EBS MEM with the addition of penicillin and streptomycin. Fecal samples were directly collected in 1 mL of HBS and EBS MEM with the addition of penicillin and streptomycin. Organ samples were transferred in 1 mL of HBS and EBS MEM with an added steel bead and homogenized at 30,000 Hz for 2 minutes with the TissueLyserII (QIAGEN, https://www.qiagen.com). Nucleic acid was extracted from 100 μL of the supernatant of all samples with the NucleoMag Vet kit (Macherey-Nagel, https://www.mn-net.com). Extracted viral RNA levels were determined by the already validated RT-qPCR nCoV_IP4, targeting the viral RNA-dependent RNA polymerase (*2*). We used a quantification cycle (Cq) value of 38 as a cutoff value (Appendix Table).

Virus Isolation

We attempted virus reisolation in cell culture on a Vero E6 cell line (L0929, collection of cell lines in veterinary medicine, Insel Riems, Germany) using HBS and EBS MEM with the addition of penicillin and streptomycin. Viral replication was determined by cytopathic effect within 72 hours after inoculation. Cultures with no visible cytopathic effect in the first passage were passaged once more.

References

1. Wernike K, Aebischer A, Michelitsch A, Hoffmann D, Freuling C, Balkema-Buschmann A, et al. Multi-species ELISA for the detection of antibodies against SARS-CoV-2 in animals. Transbound Emerg Dis. 2020;tbed.13926. PubMed https://doi.org/10.1111/tbed.13926

2. World Health Organization. Coronavirus disease (COVID-19) technical guidance: laboratory testing for 2019-nCoV in humans [cited 2020 May 16]. https://wwwwhoint/emergencies/diseases/novel-coronavirus-2019/technical-guidance/laboratory-guidance

Appendix Table. RT-qPCR results from organ samples of all inoculated and contact bank voles as well as results from the indirect, multispecies ELISA*

		RBD ELISA	RT-qPCR quantification cycle (Cq)						
dpi	Status	absorbance/result	Nasal conchae	Trachea	Lung	Bulbus olfactorius	Cerebrum	Cerebellum	Spleen
4	Inoculated	0.01/negative	25.45	33.26	37.15	32.77	34.17	32.67	Neg
	Inoculated	0.01/negative	28.23	31.53	37.32	37.05	Neg	Neg	35.21
8	Inoculated	0.86/positive	27.66	Neg	Neg	Neg	Neg	Neg	Neg
	Inoculated	0.98/positive	35.38	Neg	Neg	Neg	Neg	Neg	Neg
12	Inoculated	1.02/positive	Neg	Neg	Neg	Neg	Neg	Neg	Neg
21	Inoculated	0.93/positive	36.25	ND	ND	ND	ND	ND	ND
	Inoculated	0.39/positive	34.78	ND	ND	ND	ND	ND	ND
	Inoculated	0.60/positive	34.97	ND	ND	ND	ND	ND	ND
	Contact	0.01/negative	Neg	ND	ND	ND	ND	ND	ND
	Contact	−0.00/negative	Neg	ND	ND	ND	ND	ND	ND
	Contact	−0.00/negative	Neg	ND	ND	ND	ND	ND	ND

*RT-qPCR results are given in quantification cycle values (Cq). dpi: days postinoculation; ND, not done; Neg, negative; RBD, receptor-binding domain.
